# Echinococcosis in Tibetan Populations, Western Sichuan Province, China

**DOI:** 10.3201/eid1112.050079

**Published:** 2005-12

**Authors:** Li Tiaoying, Qiu Jiamin, Yang Wen, Philip S. Craig, Chen Xingwang, Xiao Ning, Akira Ito, Patrick Giraudoux, Mamuti Wulamu, Yu Wen, Peter M. Schantz

**Affiliations:** * Sichuan Centers for Disease Control and Prevention, Chengdu, Sichuan Province, People's Republic of China; † University of Salford, Salford, United Kingdom; ‡ Asahikawa Medical College, Asahikawa, Japan; § World Health Organization Collaborating Centre for the Prevention and Treatment of Alveolar Echinococcosis, Université de Franche-Comté, Besancon, France; ¶ Centers for Disease Control and Prevention, Atlanta, Georgia, USA

**Keywords:** Tibetan herders, alveolar echinococcosis, cystic echinococcosis, prevalence, research

## Abstract

We screened 3,199 people from Shiqu County, Sichuan Province, China, for abdominal echinococcosis (hydatid disease) by portable ultrasound combined with specific serodiagnostic tests. Both cystic echinococcosis (CE) (*Echinococcus granulosus* infection) and alveolar echinococcosis (AE) (*E. multilocularis*) were co-endemic in this area at the highest village prevalence values recorded anywhere in the world: 12.9% were infected with one or the other form (6.8% CE and 6.2% AE). Prevalences of both CE and AE were significantly higher in female than male patients and increased with the age of the person screened. Pastoral herdsmen were at highest risk for infection (prevalence 19.0%). Prevalence of CE varied in 5 townships from 0% to 12.1%, whereas AE prevalence ranged from 0% to 14.3%. Risk factors associated with both infections included the number of owned dogs, frequency of contact with dogs, and sources of drinking water.

## Abstract 2

This area has the highest prevalences of both forms of this disease in the world.

Human cystic echinococcosis (CE), caused by infection with the larval stage of *Echinococcus granulosus*, and alveolar echinococcosis (AE), caused by infection with the larval stage of *E. multilocularis*, are 2 of the most pathogenic zoonotic parasitic helminthic infections of humans in the Northern Hemisphere (*1*). Human CE occurs worldwide in association with herding, within which the main dog-sheep cycle for *E. granulosus* is transmitted (*1*). Human AE is a much rarer parasitic infection; transmission occurs in several regions of the Northern Hemisphere, including the United States, Europe, Central Asia, Siberia, Japan, and China (*2*). In China, echinococcosis occurs mainly in western regions and provinces, including Xinjiang Uygur Autonomous Region, Qinghai Province, Gansu Province, Ningxia Hui Autonomous Region, and Sichuan Province (*3*). A previous pilot survey showed that human echinococcosis was prevalent in western Sichuan Province, situated on the eastern Tibetan Plateau, and that both human CE and AE were present. The average prevalence was 4.0%; CE accounted for 2.1% and AE 1.9% (*4*).

Shiqu County (longitude 97°20´00´´–99°15´28´´E and latitude 32°19´28´´–34°20´40´´N) is located in the northwest corner of Ganzi Prefecture in Sichuan Province (average altitude 4,200 m). The county covers 25,141 km^2^, located on the eastern part of the Tibetan Plateau. Grassland covers 83.5% of this treeless area, where the weather is cold (annual average temperature –1.6°C). Ethnic Tibetans comprise 98% of the total population; they are primarily involved with livestock production and herding. The total number of livestock is >630,000. In addition, a large number of dogs, including owned dogs and strays, exist in the area (*5*). We conducted a village-based community epidemiologic study of human echinococcosis from 2000 to 2002 in Shiqu County, Ganzi Tibetan Autonomous Region, Sichuan Province, to further understand the epidemiology of human AE in this region.

## Materials and Methods

**Figure 1:**
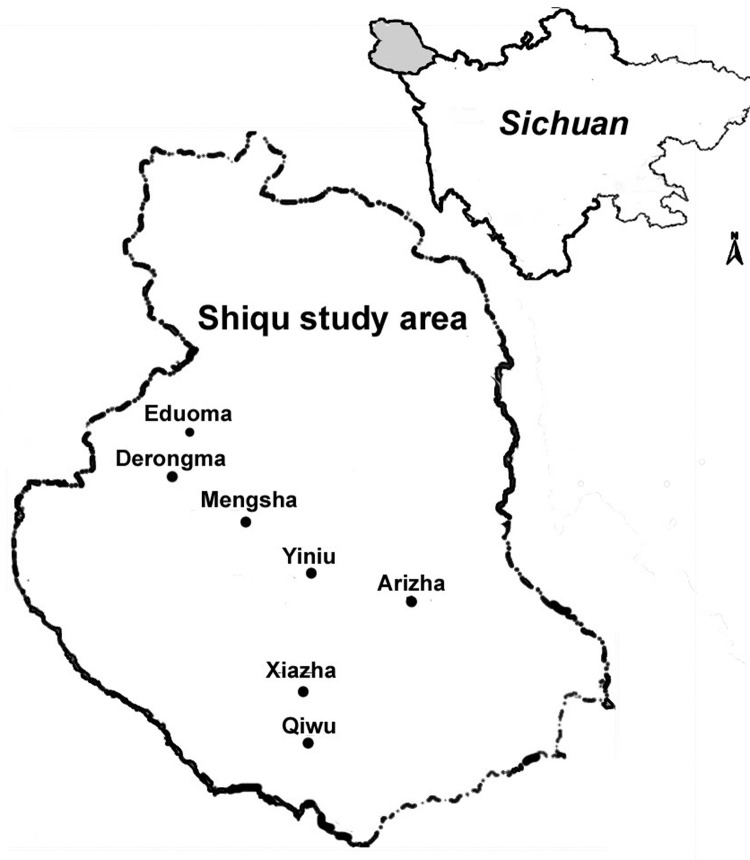
Study area in Sichuan Province, China.

The screening program was undertaken from 2000 to 2002; 26 villages in 5 townships in Shiqu County, were included (Figure 1). A total of 3,199 volunteers were self-selected after the purpose of the study was explained to the communities by local village leaders; volunteers were assured free diagnosis and chemotherapeutic treatment for echinococcosis, if indicated. Study participants ranged in age from 1 to 86 years (median 32 years). Fifty-two percent (1,660) were female patients, and 48% (1,539) were male patients. Persons of Tibetan ethnicity comprised 95% of the sampled population. The other participants listed their ethnicity as Han (4.5%), Hui (0.2%), or other (0.3%). Almost half of the participants (52.9%) raised livestock, including yaks, sheep, or goats, as the primary source of their income. Other listed occupations included student (19.1%), public servant (9.8%), preschooler (3.2%), illiterate child (2.0%), semifarmer (2.5%), farmer (1.1%), employee (2.2%), or other (7.3%).

### Questionnaire

For each registered participant, a questionnaire designed to obtain information on demographics and animal ownership was completed. Questions mainly concerned occupation, education level, dog ownership and number, frequency of dog contact, fox hunting, drinking water source, and hygienic practices.

### Screening and Diagnostic Criteria for Echinococcosis

**Figure 2:**
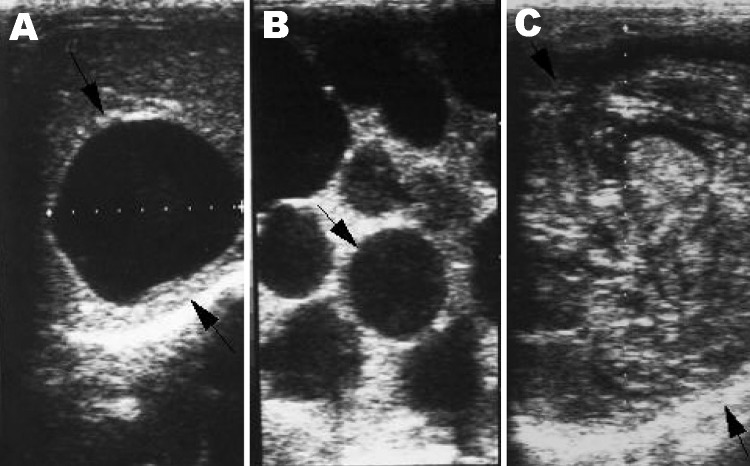
Lesions of cystic echinococcosis (CE) by abdominal ultrasound examination. A) CE lesion with distinct rim. B) Typical CE lesion with daughter cysts. C) Calcified CE lesion after chemotherapy.

**Figure 3:**
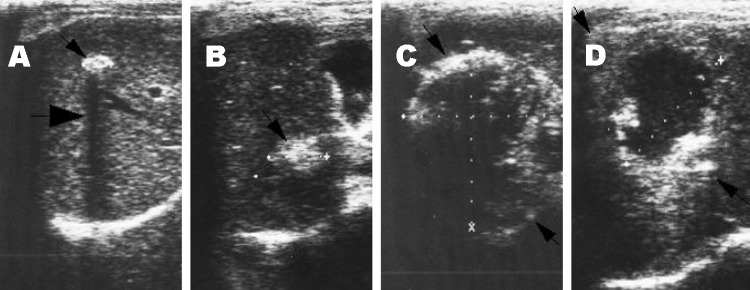
Lesions of alveolar echinococcosis (AE) by abdominal ultrasound examination. A) Calcified lesion: hyperechoic structure with a typical posterior shadow. B) Nodular hyperechoic lesion. C) Typical AE lesion: nonhomogeneous hyperechoic partially calcified area, without central necrosis. D) Typical AE lesion with central necrosis.

All participants were examined by abdominal ultrasound; those with space-occupying lesions in the liver were asked to give venous blood samples to detect *Echinococcus* antibody by using enzyme-linked immunosorbent assay (ELISA) and immunoblot with *E. granulosus* hydatid cyst fluid as antigen (*6**–**8*), as well as specific antibodies against *E. multilocularis* using ELISA and immunoblot with recombinant Em18 antigen (*9**,**10*). Diagnosis of human echinococcosis is mainly dependent on pathognomonic ultrasound images complemented by serum antibody confirmation of suspect CE/AE images (*6**,**11*). Investigators used the criteria for classification proposed by the World Health Organization Informal Working Group on Echinococcosis for CE (*11*), and the PNM system for classification of human AE, in which P stands for hepatic location of the parasite, N refers to extrahepatic involvement of neighboring organs, and M stands for absence or presence of distant metastases (*12*). CE Cases were defined as follows: 1) presence of characteristic cystlike images detected on abdominal ultrasound and a positive ELISA result with hydatid cyst fluid antigen; 2) presence of pathognomonic cyst images detected on abdominal ultrasound, but negative by ELISA (Figure 2). In addition, CE cases, on the basis of the conformational features of cysts, were differentiated into 6 types (CL, CE1, CE2, CE3, CE4, and CE5 ) and subdifferentiated by size into 3 subtypes (small [s], medium [m], and large [l]) within each type. A case of AE was defined as follows: 1) presence of pathognomonic progressive AE type lesion detected on abdominal ultrasound, regardless of serologic results; 2) presence of calcified lesions, 1–3 cm in diameter, or nodular hyperechoic lesions detected on abdominal ultrasound and seropositive against recombinant Em18; and 3) presence of a calcified lesion (1–3 cm in diameter) detected by abdominal ultrasound and negative for antibodies to the recombinant Em18 antigen but positive by ELISA, with hydatid cyst fluid (Figure 3).

### Statistical Analysis

All analyses were performed by using EpiInfo version 5.01a (Centers for Disease Control and Prevention, Atlanta, GA, USA). Statistical significance was set at p<0.01.

## Results

In this study, 84 (2.6%) of 3,199 participants examined by abdominal ultrasound scanning were determined to have an intrahepatic mass with a nonhomogeneous hyperechoic structure that contained scattered calcifications, and with irregular, poorly defined edges. A central necrotic cavity with a hypoechoic pseudoliquid structure and irregular borders was observed in 79 (2.5%) additional persons. In 53 of these participants the infiltrative lesions measured >10 cm in diameter and invaded or surrounded vascular structures, biliary structures, or both. In the other 26 persons, the lesions were nodular, were 3–5 cm in diameter at the longest dimension, and had calcifications. Calcified lesions, 1–3 cm in diameter, were observed in 20 persons. Thus, 163 persons were confirmed by ultrasound scanning to have AE infection, and 46 were suspected of having AE. Confirmatory serodiagnostic tests were performed in Japan and China, respectively. Serodiagnosis with the EgCF antigen in ELISA was positive in 93 of 94 persons with typical images of AE, 24 of 25 persons with nodular lesions, and 11 of 20 persons with calcified lesions. Additional serologic testing with the rEm18 antigen in ELISA and immunoblot was positive in 101 of 102 persons with typical images of AE, 16 of 25 with nodular lesions, and 8 of 14 with calcified lesions (Table 1). Therefore, positive confirmative serology in 35 study participants with a suspect AE image of a nodular lesion or calcified lesion indicated infection with AE. Another patient with a suspect AE image of a nodular lesion in the liver refused to give venous blood, so confirmative serologic tests could not be performed on him, and this case was not counted in the AE category. Thus, of 46 study participants with a suspect AE image, 35 were finally diagnosed as having AE. A total of 198 (6.2%) of 3,199 persons studied were determined to be infected with AE on the basis of abdominal ultrasound images and confirmatory serologic results; 15 (38.5%) of 39 infected persons had inactive, or abortive AE lesions. Ninety-five single AE lesions were located in the right hepatic lobe, and 31 were in the left hepatic lobe. Involvement of both right and left hepatic lobes by a single lesion was observed in 17 patients. In 55 cases, >2 distinct foci were observed.

**Table 1:** Serologic results for screened study participants with a suspected lesion of alveolar (AE) or cystic (CE) echinococcosis at ultrasound examination*

Ultrasound image	No. cases	Serology with rEm18		Serology with EgCF†	
		No. tested sera	No. positive sera	No. tested sera	No. positive sera
Typical image of AE‡	163	102	101	94	93
Image of suspected AE					
Nodular lesion	26	25	16	25	24
Calcified lesion	20	14	8	20	11
Image of CE					
CL	10	9	0	8	5
CE1	75	42	4	60	55
CE2	54	25	3	38	38
CE3	23	18	3	16	16
CE4	48	26	2	34	26
CE5	6	3	0	5	5
Total	425	264	137	300	273

*27.5% of study population refused to provide blood samples for serology. The data only include those study participants with a suspected lesion of AE or CE; other abnormal findings observed at hepatic ultrasound examination, such as hemangioma, biliary cyst, and gallstone, are not presented.

†EgCF, *Echinococcus granulosus* hydatid cyst fluid.

‡Typical image of AE is a nonhomogeneous, hyperechoic structure with or without a central necrotic cavity.

In addition, an ultrasound image of CE in the liver was detected in 216 (6.8%) of 3,199 study participants examined. In 10 cases, ultrasound images showed unilocular, cystic lesions with uniform anechoic content, without visible cyst wall, all <5 cm; they were considered to be type CLs. Images characterized by unilocular, simple cyst with uniform anechoic content and visible cyst wall, some exhibiting a snowflake image (7 images <5 cm, 42 images ranging from 5 to 10 cm, and 26 images >10 cm) were observed in 75 patients; they were determined to be Type CE1(7 CE1s, 42 CE1m, 26 CE1l); In 54 patients, images exhibited multivesicular or multiseptate cysts with a wheel-like appearance; others displayed unilocular cysts with daughter cysts with a honeycomb appearance. Eight of these images were <5 cm, 16 images were 5–10 cm, and 30 images were >10 cm; all of these images belonged to type CE2 (8 CE2s, 16 CE2m, 30 CE2l). In 23 cases, images were characterized by anechoic content with detachment of laminated membrane from the cyst wall, visible as a water-lily design; some had a unilocular cyst containing daughter cysts, but the whole cyst form was less rounded. Five of these cysts were <5 cm, 13 cysts were 5–10 cm, and 5 cysts were >10 cm; all were confirmed to be type CE3 (5 CE3s, 13 CE3m, 5 CE3l). In 48 cases, cysts had hyperechoic degenerative contents without daughter cysts. Seventeen of these cysts were <5 cm, 19 were 5–10 cm, and 12 cysts were >10 cm; these images belonged to type CE4 (17 CE4s, 19 CE4m, 12 CE4). Cysts characterized by thick, calcified walls in an arch-shaped form with a cone-shaped shadow, were observed in 6 cases; 3 had images <5 cm, and 3 had cysts 5–10 cm in size; these were determined to be type CE5 (3 CE5s, 3 CE5m). In 18 cases, >1 cystic lesions were identified in the abdominal cavity in addition to the liver cysts. In 5 cases, additional cysts were found in the spleen; in 3 cases, additional cysts were found in the pelvic cavity; and in l case, a cyst was also found in the kidney. Serologic results in these study participants with CE at ultrasound examination are shown in Table 1. Serodiagnosis using the EgCF antigen in ELISA was negative in 16 of 161 persons with CE; 12 of 123 persons with CE were seropositive with rEm18 by ELISA and immunoblot (Table 1). No mixed infections were observed.

### Distribution by Sex and Age

**Figure 4:**
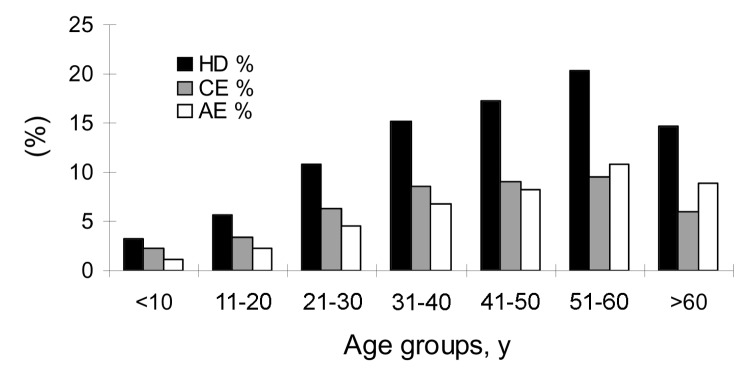
Human prevalences of echinococcosis by age groups. HD, hydatidosis; CE, cystic echinococcosis; AE, alveolar echinococcosis.

**Figure 5:**
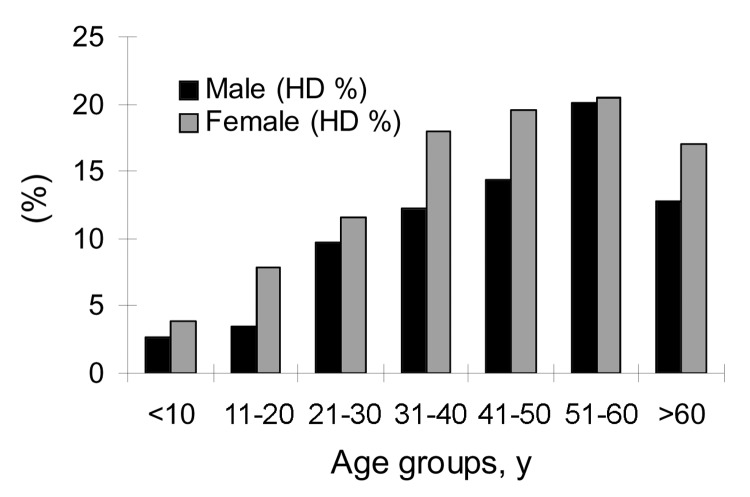
Prevalences of echinococcosis by sex and age groups. HD, hydatidosis.

Of 414 persons with evidence of abdominal echinococcosis, 244 (CE = 134, AE = 110) were female patients, and 170 (CE = 82, AE = 88) were male. Thus, the prevalence of echinococcosis in female patients was 14.7% (244/1,660), and 11.0% (170/1,539) in male patients. Thus, prevalence in female patients was significantly higher than in males (χ^2^ = 9.46, p<0.01). Compared with other older groups, the population <20 years of age had a lower infection prevalence (5.4%). In general, prevalence increased with age and reached a peak in the age group of >50 to <60 (Figure 4). The prevalence in the age group of >10 to <20 years was significantly lower than in the age group of >20 to <30 years (χ^2^ = 10.20, p<0.01). The youngest person infected with CE was 4 years of age, the oldest one was 79 years, and the average age of persons with CE was 39.0 years (n = 216). The youngest persons with AE was 8 years of age, the oldest 80 years, and the average age of AE patients was 43.1 years (n = 198) (Figure 5).

### Village Prevalence

In this study 2,033 persons were screened for echinococcosis from 26 villages in the townships of Yiniu, Mengsha, Arizha, Xiazha, and Qiwu within Shiqu County; 226 infected cases were detected. The overall township prevalence of echinococcosis was 11.1% (range 7.4%–15.1%); 6.2% of patients were diagnosed with AE and 4.9% with CE disease. The highest village prevalences for AE and CE were 14.3% and 12.1%, respectively (Table 2).

**Table 2:** Prevalence of echinococcosis determined by abdominal ultrasound in 26 villages, Shiqu County*

Township	Village	No. examined	AE	CE	Total
			No. cases (%)	No. cases (%)	No. cases (%)
Yiniu	Benri1	88	9 (10.2)	3 (3.4)	12 (13.6)
	Benri2	77	4 (5.2)	0	4 (5.2)
	Benri3	57	5 (8.8)	0	5 (8.8)
	Jiefang1	64	5 (7.8)	4 (6.3)	9 (14.1)
	Jiefang2	83	11 (13.3)	2 (2.4)	13 (15.7)
	Yiniu1	137	12 (8.8)	6 (4.4)	18 (13.1)
	Yiniu2	87	10 (11.5)	1 (1.1)	11 (12.6)
	Subtotal	593	56 (9.4)	16 (2.7)	72 (12.1)
Mengsha	Mengsha1	52	3 (5.8)	6 (11.5)	9 (17.3)
	Mengsha2	39	0	2 (5.1)	2 (5.1)
	Mengsha3	33	2 (6.1)	2 (6.1)	4 (12.1)
	Xinrong1	95	12 (12.6)	7 (7.4)	19 (20.0)
	Xinrong2	22	1 (4.5)	1 (4.5)	2 (9.1)
	Xinrong3	30	2 (6.7)	3 (10.0)	5 (16.7)
	Subtotal	271	20 (7.4)	21 (7.7)	41 (15.1)
Arizha	Arizha1	48	1 (2.1)	3 (6.3)	4 (8.3)
	Arizha2	33	2 (6.1)	4 (12.1)	6 (18.2)
	Arizha3	59	5 (8.5)	0	5 (8.5)
	Arizha4	62	5 (8.1)	1 (1.6)	6 (9.7)
	Arizha5	35	5 (14.3)	0	5 (14.3)
	Arizha6	44	3 (6.8)	2 (4.5)	5 (11.4)
	Arizha7	42	3 (7.1)	1 (2.4)	4 (9.5)
	Subtotal	323	24 (7.4)	11 (3.4)	35 (10.8)
Xiazha	Xiazha	266	10 (3.8)	13 (4.9)	23 (8.6)
	Ase	104	5 (4.8)	6 (5.8)	11 (10.6)
	Taxu	153	8 (5.2)	12 (7.8)	20 (13.1)
	Subtotal	523	23 (4.4)	31 (5.9)	54 (10.3)
Qiwu	Qiwu	219	2 (0.9)	16 (7.3)	18 (8.2)
	Getuo	78	0	3 (3.8)	3 (3.8)
	Juewu	26	1 (3.8)	2 (7.7)	3 (11.5)
	Subtotal	323	3 (0.9)	21 (6.5)	24 (7.4)
	Total	2033	126 (6.2)	100 (4.9)	226 (11.1)

*Calculations of village prevalence were based on a lower number of participants than the total study population because 1,166 study participants, including public servants, teachers, businessmen working in the area, and additional herdsmen from other townships in the vicinity of Shiqu County also participated in this survey. AE, alveolar echinococcosis; CE, cystic echinococcosis.

### Other Risk Factors

Occupation was a major risk factor. Herdsmen had the highest risk for echinococcosis infection, with a total prevalence of 19.0% (322/1,692, p<0.01); the AE prevalence was 9.5% (160/1,692), and the CE prevalence was 9.6% (162/1,692). Part-time herdsmen had a 12.7% prevalence of echinococcosis. Students and preschool children had a lower prevalence (2.8% and 3.0%), while illiterate adolescents were more heavily infected (14.3%) (χ^2^ = 21.17, p<0.01) (Table 3).

**Table 3:** Human prevalence of echinococcosis by patient occupation*

Occupation	No. examined	CE	AE	Total
		No. cases (%)	No. cases (%)	No. cases (%)
Herdsman	1,692	162 (9.6)	160 (9.5)	322 (19.0)
Parttime herdsman	79	8 (10.1)	2 (2.5)	10 (12.7)
Farmer	35	0	1 (2.9)	1 (2.9)
Student	611	8 (1.3)	9 (1.5)	17 (2.8)
Public servant	315	11 (3.5)	9 (2.9)	20 (6.3)
Employee	69	3 (4.3)	0	3 (4.3)
Businessman	17	1 (5.9)	1 (5.9)	2 (11.8)
Preschooler	101	3 (3.0)	0	3 (3.0)
Illiterate child	63	8 (12.7)	1 (1.6)	9 (14.3)
Others	217	12 (5.5)	15 (6.9)	27 (12.4)
Total	3,199	216 (6.8)	198 (6.2)	414 (12.9)

*CE, cystic echinococcosis; AE, alveolar echinococcosis.

A total of 2,811 of 3,199 persons examined answered the question about dog ownership. Of these, 496 said they did not own dogs; 2,315 (82.4%) persons had various numbers of dogs (range 1–9). Analysis indicated that the population without owned dogs had a total echinococcosis prevalence of 8.3% (41/496) (CE = 4.4%, AE = 3.8%). In contrast, persons who owned dogs had a total echinococcosis prevalence of 15.6% (360/2,315) (CE = 8.0%, AE = 7.5% [Table 4]).

**Table 4:** Human prevalence of echinococcosis by patient ownership of dogs, Sichuan Province, China*

No. owned dogs	No. examined persons	No. cases (%)		
		CE	AE	Total
0	496	22 (4.4)	19 (3.8)	41 (8.3)
1	889	67 (7.5)	65 (7.3)	132 (14.8)
2	835	61 (7.3)	66 (7.9)	127 (15.2)
3	414	38 (9.2)	29 (7.0)	67 (16.2)
>4	177	19 (10.7)	14 (7.9)	33 (19.2)
Total	2,811	207 (7.4)	193 (6.9)	400 (14.2)

*CE, cystic echinococcosis; AE, alveolar echinococcosis.

To a certain extent, education can determine occupation choice and lifestyle. Our results implied that prevalence of echinococcosis had some relationship with the level of education. Among herdsmen, 1,469 (86.8%) of 1,692 were illiterate; the prevalence in this subgroup reached 20.0% (293/1,469), the highest rate in the sampled population. The prevalence in self-identified literate herdsmen was 13.0% (29/223). Among illiterate adolescents, 14.3% were infected. Persons with only primary school education had a 6.0% (53/882) combined infection prevalence, and those with middle school education 9.1% (29/318). Persons with university education had an infection rate of 6.3% (17/268), and preschool children had an echinococcosis infection prevalence of 2.9% (3/105).

Fox hunting was also a risk factor. A total of 2,841 of 3,199 persons examined replied to the question about fox hunting. Results showed that the total prevalence of echinococcosis in populations who said that they neither hunted foxes nor kept fox skin products was 7.6% (29/384) (AE = 3.4%, CE = 4.2%), compared to a prevalence of 15.2% (368/2,427) (CE = 7.8% and AE = 7.4%) for persons who said they kept fox skin products that they had purchased, and 10% (3/30) (CE = 3 and AE = 0) in persons who said they kept fox skin products that they obtained by hunting.

## Discussion

In this mass screening study of Tibetan communities, portable ultrasound examination combined with specific serologic tests was used for the diagnosis of both CE and AE. Survey results indicated that human echinococcosis is a serious public health problem for the inhabitants of this area, for whom a 12.9% overall prevalence was recorded. In comparison with reports on human echinococcosis in other areas, including other areas of China, the prevalence in northwest Sichuan Province was much higher for both CE and AE (*1**,**3**,**12**,**13*). The prevalence of CE was higher than in other recognized echinococcosis-endemic areas of the world, including North Africa, South America, Russia, and the Middle East (1,12,14,15). Previous ultrasound-based surveys for human AE have shown regional prevalences of <0.05% in continental Europe to 4% in Gansu Province in central northwest China (*16**,**17*). The most striking observation, however, was that both AE and CE were co-endemic in this area of Sichuan, with a prevalence of 6.8% for CE and 6.2% for AE. Only parts of Turkey, Central Asia, and Siberia have been identified as co-endemic for both human CE and AE (*1**,**14*).

In Shiqu County, China, analysis of human CE and AE indicated that prevalence of disease in female patients was significantly higher (14.7%) than in male patients (11.0%). According to traditional Tibetan custom, women are usually responsible for home chores, including feeding dogs, collecting yak dung for fuel, and milking livestock. Thus, women and girls may have more opportunity to be exposed to *Echinococcus*-infected dogs and the contaminated environment.

The infection prevalence for both CE and AE for persons in the age groups <20 years was markedly lower than those of other age groups. Prevalence reached a peak among the >50- to 60-year age group. The presence of CE or AE in persons as young as 4 and 8 years, respectively, indicates recent active transmission. In general, CE or AE infection increased with age. However, among persons >60 years of age prevalence of both AE and CE declined, a situation consistent with previous reports (*4**,**15**,**18*); this finding may be associated with early death of persons infected with forms of echinococcosis, particularly with AE. A recent analysis of the relative health impact of echinococcosis in these Tibetan communities showed that CE and AE caused an average of 0.8 disability-adjusted life years lost per person (*19*), which is an exceptional value.

This analysis showed that AE infection varied from 0% to 14.3% by village and that CE village prevalence ranged from 0% to 12.1%. A trend of gradual decrease in AE in villages from north to south (9.4% vs. 0.9% in the 5 townships surveyed) was observed.

Several factors may contribute to the high prevalence of human AE in this Tibetan population. High densities of small mammals are essential to maintaining the transmission cycle of *E. multilocularis*, and small mammal populations are also subject to ecologic changes, such as deforestation or pasture overgrazing (*16**,**20**–**22*). The involvement of dogs as well as foxes in transmission in eastern Tibet, together with lack of hygiene and probable contamination of the local peridomestic environment, seem to be additional major factors (*23**,**24*). For the 5 townships located in the central area of Shiqu County, the geographic conditions, apparent ecologic factors, life style, religion, livestock production, and dog ownership practices appear to be similar; however, human AE village prevalence was markedly variable. We had previously observed that local differences in small mammal abundance over time, possibly associated with overgrazing practices may contribute to variable township AE disease rates (*22*).

This survey disclosed that 86.8% of herdsmen were illiterate; 20% of them had either CE or AE disease. Consequently, improving the knowledge and awareness of the disease among the traditional nomadic population is imperative in any future control or prevention studies. Analysis indicated that both CE and AE risk was related to dog ownership (p<0.01), contact with dogs (p<0.01), source of drinking water, and general hygiene (p<0.01). While the role of domestic and working dogs as the major definitive host for *E. granulosus* is clear, such is not the case for *E. multilocularis*. Of particular interest therefore was the strong association between human AE risk and dog ownership or contact. Evidence from community studies in other parts of China (16), the United States (*25*), and Germany (*26*) increasingly show that the domestic dog plays a key role in the zoonotic risk for human AE.

Dogs are kept in large numbers by Tibetans and are used primarily to guard property and livestock. In this survey, 82.4% of the population owned dogs, and 21% owned >3 dogs. Buddhist practice forbids killing any animal, including dogs, and this practice leads to large numbers of stray dogs, which mainly gather around temples or townships, where they are fed by monks and herdsmen. Dogs also are predators of small mammals on adjacent pastures; these dogs are usually fed by herdsman with offal (including liver and lungs) of sheep and yaks during slaughtering season. Necropsy of intestines of stray dogs in 1995 in this region showed a 29.5% prevalence for *E. granulosus* and 11.5% for *E. multilocularis* (*27**,**28*). A recent diagnostic purgation study of dogs in this area demonstrated *E. multilocularis* prevalence of 12% and an *E. granulosus* prevalence of 8% (*29*). Foxes are the main sylvatic hosts of *E. multilocularis*, and both the Tibetan fox (*Vulpes ferrilata*) and the red fox (*V. vulpes*) are common on the Qinghai-Tibet plateau. A previous report showed a high prevalence of *E. multilocularis* in the Tibetan fox (59.1%) and red fox (57.1%) (28) in this area. Furthermore, Qiu et al. observed in 1995 the existence of *E.* strobilae in Tibetan foxes with morphologic characteristics distinct from *E. multilocularis* adults but considered it to be a variant of *E. multilocularis*. These specimens and new samples have been shown to be a new species of taeniid cestode belonging to the genus *E.* Rudolphi (*30*). However, whether the new species is involved in the transmission of a third form of human echinococcosis in this region has yet to be determined.
